# Pre-hospital Care to Trauma Patients in Addis Ababa, Ethiopia: Hospital-based Cross-sectional Study

**DOI:** 10.4314/ejhs.v31i5.14

**Published:** 2021-09

**Authors:** Tsegaye G/Ananya, Menbeu Sultan, Biruktawit Zemede, Ayalew Zewdie

**Affiliations:** 1 Adigrat University, Ethiopia; 2 St Paul's Hospital Millennium Medical College, Addis Ababa, Ethiopia

**Keywords:** Pre-hospital care, Trauma, Emergency department, Ambulance

## Abstract

**Background:**

Trauma is a major cause of morbidity and mortality worldwide. Prompt use of pre-hospital care is associated with reduced early and late morbidity and mortality from trauma. This study aimed to assess the time to reach the facility and the pattern of pre-hospital care provided for trauma patients.

**Methods:**

A cross-sectional study design with a structured interview questioner was used for patients presenting to Addis Ababa Burn Emergency and Trauma Hospital Emergency Department from April 1 to May 30, 2020.

**Result:**

Out of 238 interviewed patients, the most common means of transportation from the scene to the initial health facility were taxi 77(32.4%) and ambulance 54(22.7%). The time of arrival from the scene to the initial health care facility was within one hour, 133(56.1%) and in 1–3 hours 84(35.5%). Some form of care was provided at the scene in 110(46.2%) of cases. The care provided was bleeding arrest 74(31.1 %), removing from wreck 51(21.4%), splinting/immobilizing injured area 38(16%), position for patient comfort 19(8%), and others. Relatives were the most common care provider 49(45%) followed by bystanders 37(33.9%), trained ambulance staff 19(17.4%), and police 2 (1.8%). The main reasons for not providing care were lack of knowledge 79(61.2%), and lack of equipment 25 (19.4%).

**Conclusion:**

The study showed relatives and bystanders were the first responders during trauma care. However, ambulance utilization for pre-hospital care was low. There was trauma patients delay to arrive to hospital. Only half of the patients presented to the health facility within Golden hour.

## Introduction

Injury is a major cause of early death and disability worldwide. Every year approximately 5 million people die in the world. Deaths from severe injury occur in one of three phases: Immediate (death occurring from overwhelming injury), intermediate or subacute (death occurring within several hours of trauma from treatable conditions), and delayed (death occurring days to weeks after trauma as a result of infection, multi-organ failure or other late complications) (1–3).

The most effective way to prevent mortality and morbidity from trauma is the prevention of occurrence of trauma but providing effective pre-hospital care minimizes morbidity and mortality. Most deaths in the first hours of trauma are airway obstruction, hypoxia, and hemorrhage all of which can be reduced using effective first aid measures(1,4).

Prompt pre-hospital care can also prevent delayed death of trauma patients with proper wound and burn care, immobilization of fractures, and support of oxygen and blood pressure in traumatic brain injury. Pre-hospital care reduces mortality and morbidity from serious illness and injuries. It is estimated that about 45% of mortality and 35% morbidity can be reduced by providing robust out of hospital emergency care(2).

In low and middle-income countries without formal emergency care system around 80% of deaths in severe trauma occurred in pre-hospital setting(5, 6). In Ethiopia in the past 10 years, the Federal Ministry of Health has introduced an effort to improve the Emergency Medical Service (EMS) systems. Efforts include distribution of ambulances to all regions, providing at least one ambulance per district (woreda), training of paramedics, and procurement of on-board medical equipment. In Ethiopia, there is less grown pre-hospital care and a paucity of study on the existing level of care and determinants of care(4,7).

This study aimed to assess the time to reach the facility and the pattern of pre-hospital care provided for trauma patients visiting a tertiary care trauma center in Addis Ababa.

## Methods

**Study area period**: This study was conducted from April 1 to May 31, 2020 at Addis Ababa Burn Emergency and Trauma (AaBET) Hospital. AaBET Hospital is a part of St. Paul's Hospital Millennium Medical College. It is an emergency dedicated center with level 3 trauma care. It provides emergency and critical care services, Orthopedic, Neuro-surgery, General surgery, and Plastics surgery services. The emergency department has 60 beds and the overall hospital bed is 300. Annual emergency room patient visits ranges from 15,000 to 20,000.

**Design and sampling**: A hospital-based cross-sectional study design was used. The source population was all trauma patients seen in Addis Ababa Burn Emergency and Trauma (AaBET) Emergency Department (ED). All adult (age >15 years) trauma patients who came to AaBET Hospital Emergency Department during the 2 months study period were included. Patients sent to ED from regular Out Patient Department (OPD) for admission, death on arrival, and patients who came for follow-up were excluded

**Sample size determination**: Sample size calculated by using the single population proportion formula, prevalence of (0.17) was used where 17 % of patients received pre-hospital care (4), with 10% error sample size of 238 patients was included using simple random sampling from a total of 1064 trauma patients seen during the 2 month study period.

**Data collection and analysis**: Data was collected using a pilot-tested, structured, interviewing questionnaire prepared in English and translated to the patient's mother tongue language by two data collectors from the patient and/or care givers. The questionnaire was prepared by reviewing different kinds of literature and undertaking modifications for the population studied. (4, 7) The data collectors were trained about the data collection and the quality of data was check by the principal investigator. Data collection was done after patient clinical stabilization. Patients in critical condition and/or unable to communicate data was collected from care givers.

Data were cleaned initially before entering to analysis using SPSS version 21. Descriptive statistics were done on demographics, mode of transport, mechanism of transport, body site of injury, and time of arrival to the hospital.

Associations were done using the chi-square test and binary logistic regression was used to determine factors associated with pre-hospital care provision. All tests with P-Value< 0.05 was considered statistically significant. Tables and graphs were used for data presentation.

**Ethics Approval**: The research proposal was approved by St Paul's Hospital Millennium Medical College's research ethical committee. This study was conducted per the Declaration of Helsinki: each study participant was well informed about the aim of the study, benefits, and risks; informed written consent was secured from study participants; study participants' confidentiality was maintained; no personal identifiers were used in the data collection questionnaire, and codes were used in place of them. Emergency care intervention was not delayed for the interview.

## Results

**Socio-demographic characteristics**: Out of 238 included patients, 186(78.2%) were male with a male to female ratio of 3.6:1. The mean age was 32.25 years with an SD of 13.45years. Most patients were from Oromia 117(49.2%) and Addis Ababa 104(43.7%). Regarding occupation, the majority were day laborers 81(34%) followed by farming 40(16.8%). (Table 1).

**Table 1:** Socio-demographic characteristics and time to a first health facility of trauma patients presented to AaBET Hospital, Addis Ababa, Ethiopia, April 1 – May 30, 2020

Variable	Alternatives	Frequency	Percent (%)
	Female	52	21.8
Age(in years)	≤30	160	67.2
	31–45	58	24.3
	>45	30	8.5
	Mean age	32 ± 13	
Residence/region	Oromia	117	49.2
	Addis Ababa	104	43.7
	Amhara	10	4.2
	SNNP	4	1.7
	Others	3	1.3
Occupation	Day laborer	81	34
	Farming	40	16.8
	Private office work	39	16.4
	Driver	28	11.8
	Government employee	32	13.4
	Other	18	7.6

**Clinical profile**: The most common mechanism of trauma was a road traffic accident 102(42.9%) followed by falling 62(26.1%). Trauma to extremity region 135(56.7%) was the most common site of injury followed by the head 86(36.1%). Upon arrival, patients were triaged to yellow-green 185(77.8%), 39(16.4%) orange, and 16(5.9%) red side. The majority of patients had sustained the injury at work 87(36.6%), public gathering 46(19.3%), pedestrian 44(18.4%), passenger 35(14.7%), and driving 19(8%) (Table 2).

**Table 2:** Summary of triage, mechanism of trauma, and type of trauma patients presented to AaBET Hospital ED, Addis Ababa, Ethiopia, April 1–May 30 2020

Variable	Alternatives	Frequency	Percent
Triage category	Yellow	142	59.7
	Green	43	18.4
	Orange	39	16.4
	Red	14	5.9
Mechanism of trauma	RTA	102	42.9
	Fall down	62	26.1
	Fighting(stick/stone)	39	16.4
	Stab	14	5.9
	Bullet	10	4.2
	Machine	8	3.4
	Others (burn and dog bite)	3	1.3
Type of trauma	Extremities	135	56.7
	Head	86	36.1
	Chest	20	8.4
	Abdomen	14	5.9
	Spine	19	8
Patient activity during	Workplace	87	36.6
trauma	Public gathering	46	19.3
	Pedestrian	44	18.5
	Passenger	35	14.7
	Driving	19	8
	Other	7	2.9

**Pre-hospital care**: One hundred ten patients (46.2%) received some form of pre-hospital care mainly first aid at the scene. Cares provided at the scene were positioning patient 19(8%), bleeding arrest 74(31.1 %), splinting/immobilizing injured area 38 (16%), removing from wreck 51(21.4%) and others like calling police or others for help 7 (2.9%).

Relatives were the most common care provider (49(45%)) followed by bystanders 37(33.9%), trained ambulance staff 19(17.4%), and police 2 (1.8%) provided first aid. The main reasons for not providing care were lack of knowledge 79(61.2%), followed by lack of equipment 25 (19.4%), fear of procedure 11(8.5%), fear of medico-legal issue 9(7%) and others like fear of transmitted diseases 5(3.9%).

One hundred thirty-three (56.1%) patients presented to the first health facility within one hour. 57 (33.5%) of them transported to first health facility using taxi. Time of arrival and mode of transport is shown in Table 3.

**Table 3:** Time of arrival to the first facility against the mode of transport presented to AaBET Hospital ED, Addis Ababa, Ethiopia, April 1–May 30 2020

Mode of transport		Time of arrival to a first health facility			Total (%)

		10–60 min	60–120 min	> 120 min	
Mode of transport	Ambulance	28(16.5%)	16(41.0%)	9(33.3%)	53(22.5%)
	Taxi	57(33.5%)	11(28.2%)	9 (33.3%)	77(32.6%)
	private vehicle	27(15.9%)	4(10.34%)	3(11.1%)	34(14.4%)
	carried by people	25 14.7%	4 10.3%	2 7.4%	31 13.1%
	Walking	30 17.6%	3 7.7%	4 14.8%	37 15.7%
	Others	3 1.8%	1 2.6%	0 0.0%	4 1.7%

**Inter health facility referral**: A total of 155(65.1%) were referred from different areas, only 83(53.5%) were referred with communication. The most common source of referral was public hospital 79(46.7%) followed by public health center 74(43.7%) and private facility 16(9.6%). About 142(82.6%, n= 172) were transported by ambulance, 20(11.5%, n=172) with taxi, 9(5.2%, n= 172) by private car and one patient walking.

**Determinants of pre-hospital care**: Chi-square test was used to assess the association between socio-demographic, mode of transport, mechanism of trauma, and type of trauma with the delivery of pre-hospital care. Only mechanism of injury had statistically significant association with provision of pre-hospital care (P-value=0.04) (Table 4).

**Table 4:** Association between demographics and pre-hospital care for trauma patients presented to AaBET Hospital ED, Addis Ababa, Ethiopia, April 1–May 30 2020

Variables		Prehospital care			OR(CI)	P-Value

		Yes(110) n(%)	No(128) n(%)	Total N(%)		
Age	<29	70(63.6)	69(53.9)	139(58.4)	1.49(0.889–2.520)	0.13
	>29	40(36.4)	59(46.1)	99(41.6)	1.0	
Sex	Male	86(78.2)	100(78.1)	186(78.2)	1.003(0.542–1.859)	0.99
	Female	24(21.8)	28(21.9)	52(21.8)	1.0	
Residence	Addis Ababa	54(49.1)	50(39.1)	1104(43.7)	1.504(0.889–2.519)	0.12
	Out of Addis Ababa	56(50.9)	78(60.9)	134(56.3)	1.0	
Mode of arrival	Ambulance	28(25.5)	26(20.3)	54(22.7)	1.34(0.729–2.460)	0.34
	Other means	82(74.5)	102(79.7)	184(77.3)	1.0	
Mechanism	RTA	40(36.4)	63(49.2)	103(43.3)	0.590(0.350–0.992)	0.046*
	Non-RTA	70(63.6)	65(50.8)	135(56.7)	1.0	
Type of trauma	Poly-trauma	13(11.8)	25(19.5)	39(16)	0.552(0.267–1.140)	0.11
	Not	97(88.2)	103(80.5)	200(84.0)	1.0	

* P Value<0.05 is considered as statistically significant.

## Discussion

This study showed the overall utilization of pre-hospital care for trauma patients was 110(46.2%) which showed improvement from a study done in Addis Ababa Tikur Anbesa Specialized Hospital (TASH) seven years back (16.7%), also higher than the study from India 26.5%, southwest Nigeria 8.6%(4, 8, 9). It is comparable to cross-sectional study done in Hanoi, Vietnam 48%(10). Authors suggested that this improvement could be because of community awareness and different stakeholders' engagement in decreasing trauma. However, this requires further study.

Time to the health facility is crucial factor for trauma patients. Based on The Golden hour concept one hour is determined to be determinant for patients' better survival(11). In this study, 56.1% of patients reached the initial health facility within one hour was comparable to studies done in Kenya, 66.2% arriving within one hour and southwest Nigeria with 57% arriving in the first one hour of trauma(12,13). But this time was better than a study from Tikur Anbessa showed 18.5% arrive in one hour and 57% arrive in 1–2 hours(4). The study from Seattle showed 75% arriving initial facility within thirty-minute of trauma(14). This significant variation may be related to differences in availability of the nearby facility, transport infrastructure, and community awareness of where and when to go.

The majority of scene care provisions in our study were none trained relatives; this is consistent with the reports from developing nations(4). However, in developed countries like the USA and UK studies show scene care is delivered by well-trained personnel(15,16). This variation may arise from the presence of a single call number and community awareness on utilization of ambulances. Most cares provided in our study are at their basic level arrest bleeding (33.9%), removing the patient from the trauma area (21%), immobilization (16%), and others. this is also consistent with the reports from the developing nations(4,12).

The explanation for this may be because most of the providers are none trained bystanders and relatives, unlike the developed countries where care is by personnel with advanced training and equipped ambulances. It is better to train the community on first aids via community health workers and awareness creation with mass media.

Upon analysis for means of transportation to the initial health facility, our study showed taxi was the most common means (32.4%) followed by ambulance (22.7%), walking with support (15.5%), and others. This was in keeping with studies done in Tikur Anbessa, Vietnam, and India where the majority of patients were transported by taxi and private vehicles(4,8,13). A study in Kenya showed only 1.4% ambulance use and that of Tanzania showed no patient transported by ambulance (12, 13). However, studies in Britain and the USA showed more than 96% ambulance usage(15, 16). This low utilization of ambulance for transport may be due to inadequate distribution, poor infrastructure, lack of community awareness on how to get them, and the large shift in health system resources including ambulances to fight the pandemic. Ambulances were used mostly for interfacility transfer. Taxi use was the most common mode of transport in this study and other African studies which indicates there should be a way for rethinking about ambulance-based pre-hospital care which is standard care from westerns (4, 12, 13). There should be African based solution for better pre-hospital trauma care. Training taxi drivers about trauma first aid and equipping taxis the necessary equipment will improve pre-hospital trauma care.

Although this study provided a good incite to pre-hospital care of trauma patients, it was conducted during a pandemic which jeopardized the health system shifting the majority of resources for the pandemic subsequently interfere with the pattern of some variables. This study is hospital-based, single centered making, cross- sectional and short study period making it liable for selection bias and limits generalizability.

In conclusion, this study showed relatives and bystanders were the first responders during trauma care. However, ambulance utilization for pre-hospital care was low. There were trauma patients delay to arrive to hospital. Only half of the patients presented to the health facility within Golden hour.
